# Chow diet in mouse aging studies: nothing regular about it

**DOI:** 10.1007/s11357-023-00775-9

**Published:** 2023-04-20

**Authors:** Jennifer Lee, Chloe Purello, Sarah L. Booth, Brian Bennett, Christopher D. Wiley, Ron Korstanje

**Affiliations:** 1 Jean Mayer USDA Human Nutrition Research Center On Aging at Tufts University, 711 Washington St, Boston, MA USA; 2 Agricultural Research Service, US Department of Agriculture, Western Human Nutrition Research Center, Davis, CA USA; 3 Department of Nutrition, University of California Davis, Davis, CA USA; 4 The Jackson Laboratory, Bar Harbor, ME USA

**Keywords:** Chow diet, Dietary source, Aging

## Abstract

Chow diet is used in the majority of rodent studies and, although assumed to be standardized for dietary source and nutritional contents, it varies widely across commercial formulations. Similarly, current approaches to study aging in rodents involve a single-diet formulation across the lifespan and overlook age-specific nutritional requirements, which may have long-term effects on aging processes. Together, these nutrition-based disparities represent major gaps in geroscience research, affecting the interpretation and reproducibility of the studies. This perspective aims to raise awareness on the importance of rodent diet formulation and proposes that geroscientists include detailed descriptions of all experimental diets and feeding protocols. Detailed reporting of diets will enhance rigor and reproducibility of aging rodent studies and lead to more translational outcomes in geroscience research.

**Supplementary Information:**

The online version contains supplementary material available at 10.1007/s11357-023-00775-9.

## Introduction

Rodent models of aging are critical for advancing the field of geroscience but biological heterogeneity has long hampered the reproducibility of aging rodent studies [1, 2]. It is known that diet has a major impact on aging and that dietary intake of macronutrients modulates the aging process [3, 4]. Furthermore, subtle variations in dietary composition can have substantial effects leading to variable rodent phenotypes that impact metabolism and aging-associated outcomes [2–4]. Despite its importance, the role of diet heterogeneity is particularly underappreciated when interpreting results of aging studies. We propose that enhancing awareness regarding the importance of diet among geroscience investigators and requiring more rigorous descriptions of diets in funding applications and in peer-reviewed publications will improve reproducibility in aging rodent studies. While acknowledging that the same principles apply to all animal models of aging, this review will be focused on rodent models.

## There is no such thing as a “regular chow diet”

Most peer-reviewed publications using rodent models of aging include little, if any, description of the diet used in the study design. Indeed, the majority contain terms such as “food and water” or “chow.” The assumption behind this lack of detail on the diet is that all “standard” chow diets are the same. However, careful comparison of the nutritional values of two commonly used diets such as LabDiet® 5K0G and Teklad 2018 (Table 1, Supplemental Data) reveals major differences between the two. For example, gross energy (kcal/g) is 4.18 in LabDiet® 5K0G versus 3.1 in Teklad 2018, while levels of vitamin A and vitamin D_3_ are, respectively, 4.44- and 117.3-fold greater in the former compared to the latter. By contrast, levels of vitamin E and iodine are, respectively, 1.64- and 2.8-fold greater in Teklad 2018 compared to LabDiet® 5K0G. Notably, nine dietary constituents found in LabDiet® 5K0G are absent or not reported in Teklad 2018 (taurine, arachidonic acid, omega-3 fatty acids, carotene, cholesterol, sulfur, fluoride, cobalt, and chromium; Table 1), all of which have known bioactive properties that may impact metabolism. These differences may contribute to diverse rodent metabolic phenotypes and aging outcomes, lack of reproducibility, and erroneous conclusions even in highly controlled experimental setups involving genetically identical inbred rodents.

**Table 1:**
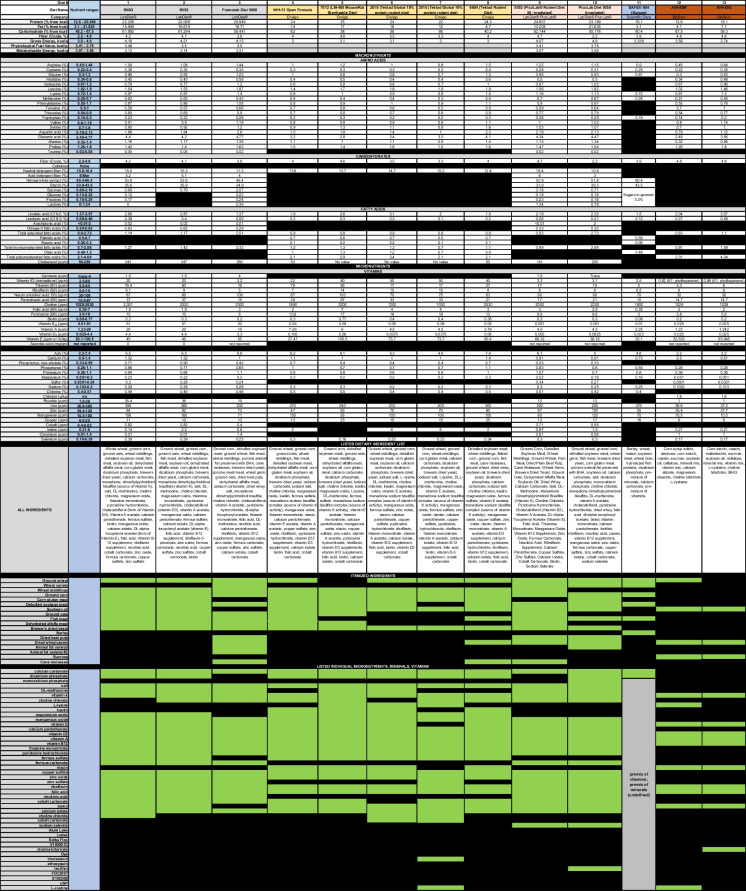
Comparison of the nutritional values of commonly used diets

Green indicates that the dietary component is present in the diet. Black indicates that the dietary component is not found in the diet

It has been long appreciated within the nutrition field that standardization of rodent research diets is critical for reproducibility of results across all disciplines [5]. Nutritional guidelines can reduce variability among rodent studies, and thus prevent “wasted effort and money caused by failure to duplicate research findings and faulty conclusions in nutritional, toxicological, behavioral, and cancer studies” [6]. While commercially produced chow diets have consistently supported growth in rodent models, their historically uncontrolled variability in minerals and vitamins has sometimes resulted in nutritional inadequacies [5]. This prompted the American Institute of Nutrition (AIN), now known as the American Society of Nutrition (ASN), to develop formula rodent diets in 1976, with clearly stated percentages and/or quantities of ingredients that met established rodent nutrient requirements at that time. This initial formulation did provide a critical framework to develop standardized diets for rodents but was not without challenges. In fact, the diet was designed to meet nutritional needs for all rodents but was found to induce ectopic calcification of the kidneys in female Sprague Dawley rats [7].

The AIN diet was revised in 1993 with a focus on weight gain over 3–4 months. The final adopted formulation(s), named AIN-93, resulted in a 13% weight increase in Swiss-Webster mice but not in Sprague–Dawley rats [8]. Of particular relevance to this commentary and the field of geroscience is the fact that these diets and their efficacy were designed to maximize bodyweight during growth of relatively young mice. Subsequent studies, which further examined the effects of micronutrient manipulation in mice, were performed on mature mice but not specifically aged mice. For example, dietary copper requirements were determined in 20-week-old male mice fed AIN-93 diets with variable levels of copper [9]. Thus, our understanding of nutrient requirements remains limited within the context of aging.

The current AIN-93 diet is assumed by many to be a “regular” chow diet. However, AIN-93 is a defined diet formulated from purified ingredients, while “chow” generally comprise a variety of grain or cereal-based diets, including soybean meal, corn, fish meal, and animal byproducts in either openly disclosed (open) or proprietary (closed) formulations (Table 1). This lack of dietary information poses issues when trying to reproduce and/or compare results among rodent studies. In addition, batch effects, which are impacted by factors including season-dependent nutritional quality of the crops, may result in distinct compositions of the diets within and across commercial manufacturers. In the absence of a direct analysis of the ingredients for each batch of diet, substantial variability in the formulation can be inadvertently introduced over the course of an aging rodent study. Diet variability can confound the results of studies that compare phenotypes of interest at different points in the lifespan, thus, heavily impacting the interpretation of research outcomes.

The different compositions of chow diets can induce a shift in macronutrient ratios that can in turn impact research results and create challenges in reproducibility across studies. For example, the carbohydrate component is 63% (10% sucrose and approximately 53% cornstarch) in the AIN-93G formula, while is 62% (cornstarch only) in the AIN-93 M formula (5). Importantly, the AIN-93G is formulated for growth, pregnancy, and lactation, whereas the AIN-93 M is formulated for maintenance. The Open Formula NIH-31 diet (62% carbohydrates from ground corn and corn gluten meal), which is the standard rodent reference diet for the National Institutes of Health (NIH), is formulated for maintenance in addition to growth, reproduction, and lactation in rodents. No diet has been developed for the specific purpose of aging, yet every nutritional component in rodent diets may have distinct biological effects that impact aging processes.

Chow diet variability extends beyond that of absolute macro- and micronutrient concentrations. In fact, although autoclaving and irradiation are intended to sterilize diets to meet animal barrier facility regulations, heat treatment (heat, pressure, steam) conditions are variable and directly affect nutrient bioavailability [10]. For example, the autoclaving process is known to alter the levels of heat-labile vitamins such as vitamin K [11], and the bioavailability of soy protein [10]. As a way to circumvent this issue, the NIH-31 Open Formula Autoclavable diet contains additional amounts of vitamins to compensate for losses upon steam sterilization (Table 1).

## What is the appropriate diet for aging rodent studies?

In the absence of systematic long-term studies on the dietary requirements of aging rodents, it has been assumed that chow diets developed for the maintenance of younger animals are also suitable for studies on aging. However, the effects of such diets on aging in mice remain unclear, largely because it is hard to discern physiological changes associated with aging (mitochondrial dysfunction, cellular senescence, and neurodegeneration) from diet-induced effects.

Long-term studies, such as The Study of Longitudinal Aging in Mice (SLAM), will ultimately provide unique insight into normative aging (1). Here, 8-week-old inbred C57BL/6 J and outbred UM-HET3 mice of both sexes were purchased from The Jackson Laboratory (Bar Harbor, ME) and fed LabDiet 5K0G (22% protein, 16% fat, and 62% carbohydrates). During 1 month of acclimatization, mice were fed Envigo 2018SX (24% protein, 18% fat, and 58% carbohydrate) before being transitioned to the open source NIH-31 formula (24% protein, 14% fat, and 62% carbohydrate) (Envigo, Table 1) for the duration of the study. In addition, fiber content varied between 4.2% in LabDiet® 5K0G, 3.5% in 2018SX, and 4% in NIH-31 (Table 1). Fiber content impacts gut microbiome function and host metabolism [12]. Although it is likely that differences in fiber levels across diets will impact aging processes [12], it is still unknown whether these multiple modifications in macronutrient ratios (and their source) during the first 3 months of life will have an impact later in life. Regardless, we commend the SLAM investigators for publishing data on the diet manufacturers and formulations as well as timelines for transition of diets, as these data will be critical when comparing aging rodent studies that use different diet formulations.

## Caloric restriction

Caloric restriction (CR), through a reduction in total diet intake, has been demonstrated to extend longevity in rodents [13, 14]. In a unique rodent study comparing longevity between ad libitum fed and dietary-restricted rats (31% less overall energy intake), Duffy et al. reported that survival rates for the ad libitum fed were lower than those of the dietary-restricted group, consistent with findings from other studies [14, 15]. Both groups of rats were fed a purified AIN-93 M diet (casein as source of protein) that had the same content of vitamins and mineral per gram of diet, such that the calorie-restricted animals had a commensurate reduction in micronutrient intake. The consequences of inadequate vitamin and mineral intake in dietary restricted rats were not analyzed in this work. The authors then compared their results to a prior rodent study in which rats were fed the cereal-based NIH-31 Open Formula, either ad libitum or at 25% or 40% dietary restriction [14]. In contrast to the AIN-93 M diet regimen which did not adjust for vitamins and minerals, the rats fed the NIH-31 Open Formula were fed a formulation that contained 1.67 × additional vitamin mix. While survival was no different between the rats that were fed either diet ad libitum, the rats fed the AIN-93 M diets at 31% dietary restriction had lower survival compared to those fed the NIH-31 formula at both 25% and 40% dietary restrictions. Although there were no differences in survival when compared to NIH-31-fed rats, ad libitum consumption of AIN-93 M by Sprague Dawley rats resulted in approximately a 10% increase in bodyweight at 96 weeks of age as compared to ad libitum consumption of NIH-31 [16]. This study highlights the importance of documenting the exact composition of diets when manipulating macronutrients to extend longevity in murine models. Of note, despite the importance of micronutrients (vitamins and minerals) in regulating metabolic processes and their likely impact on aging outcomes, alterations in their levels are generally not accounted for in CR rodent studies.

The team of LeCouteur and colleagues elegantly leveraged rodent aging studies that have complete diet information to demonstrate the importance of macronutrient ratios [3, 17]. These studies reported that female and male mice that were fed ad libitum with higher protein and lower carbohydrate diets had the greatest reproductive potential. In contrast, female and male mice fed ad libitum with lower protein and higher carbohydrate diets had longer lifespans, independent of their caloric intake. Several conclusions can be drawn from these studies. It is critical to better define and report macronutrient ratios and compositional subtypes (i.e., unsaturated versus saturated fats [18]) instead of simply reporting the calories and their degree of restriction in rodent studies. Perhaps more importantly, the fact that a protein-rich diet is beneficial during reproduction but detrimental to aging indicates that rodent diet formulations and their macronutrient ratios should be adjusted over the lifespan instead of being kept throughout, as in the current practice. Furthermore, it has been shown that circadian alignment impacts the effect of caloric restriction [19]. Therefore, it is important to include details on the exact time of feeding as related to the light cycle. Lastly, much more research is needed to determine whether it would be beneficial to adjust nutrient by age (age-specific diets).

## Looking forward

In order to improve scientific rigor and reproducibility, the geroscience research community needs to better define the composition of rodent diets used in aging studies, preferably across the lifespan. We propose two ways to enhance this awareness: (1) review process of grant applications submitted to the National Institute of Aging (NIA); and (2) review process of manuscripts submitted to geroscience journals.

NIH provides clear guidance on how to address rigor and reproducibility for all grant applications submitted, including a mandatory section entitled**, “**Consideration of relevant biological variables.” Currently, biological variables are defined as sex, age, weight, and underlying health conditions. We propose that the NIA considers diet as an additional mandatory biological variable. Applicants should be required to describe the exact diet formulations and provide details of diet modifications to be made within the study design. This will raise awareness on the importance of rodent diet formulation in research studies and improve the assessment of rigor in prior research (20).

We propose that geroscience journals that publish rodent studies require a detailed description of both the control and experimental diets. When a diet composition is published for the first time in a journal, complete information on all the components, including any modifications to published diet compositions, should be presented in a table. Nutrition journals, such as *Journal of Nutrition*, have already implemented this requirement and it is our opinion that research progress in other fields would benefit from a standardization in the reporting of this important yet overlooked experimental variable.

In summary, detailed reporting of diets in aging rodent studies will enhance reproducibility and lead to more translational outcomes, thus improving the use and relevance of animal models in geroscience research.

## Supplementary Information

Below is the link to the electronic supplementary material.

Supplementary file1 (PDF 1794 KB)

## References

[1] Palliyaguru DL, Vieira Ligo Teixeira C, Duregon E, di Germanio C, Alfaras I, Mitchell SJ (2021). Study of longitudinal aging in mice: presentation of experimental techniques. J Gerontol A Biol Sci Med Sci.

[2] Mitchell SJ, Madrigal-Matute J, Scheibye-Knudsen M, Fang E, Aon M, González-Reyes JA (2016). Effects of sex, strain, and energy intake on hallmarks of aging in mice. Cell Metab.

[3] Solon-Biet SM, McMahon AC, Ballard JW, Ruohonen K, Wu LE, Cogger VC (2014). The ratio of macronutrients, not caloric intake, dictates cardiometabolic health, aging, and longevity in ad libitum-fed mice. Cell Metab.

[4] Gibbs VK, Smith DL (2016). Nutrition and energetics in rodent longevity research. Exp Gerontol.

[5] Klurfeld DM, Gregory JF, Fiorotto ML (2021). Should the AIN-93 rodent diet formulas be revised?. J Nutr.

[6] Nielsen FH (2018). 90th anniversary commentary: the AIN-93 purified diets for laboratory rodents—the development of a landmark article in The Journal of Nutrition and its impact on health and disease research using rodent models. J Nutr.

[7] Shah BGTK, Belonje B (1986). Factors affecting nephrocalcinosis in male and female rats fed AIN-76 salt mixture. Nutr Res.

[8] Reeves PG, Nielsen FH, Fahey GC (1993). AIN-93 purified diets for laboratory rodents: final report of the American Institute of Nutrition ad hoc writing committee on the reformulation of the AIN-76A rodent diet. J Nutr.

[9] Reeves PGRK, Johnson L (1994). Maintenance requirements for copper in adult male mice fed AIN-93M rodent diet. Nutr Res.

[10] Taciak M, Tuśnio A, Święch E, Barszcz M, Staśkiewicz Ł, Skomiał J (2015). Effects of autoclaving soy-free and soy-containing diets for laboratory rats on protein and energy values determined in vitro and in vivo. J Am Assoc Lab Anim Sci.

[11] Fu X, Booth SL, Smith DE (2007). Vitamin K contents of rodent diets: a review. J Am Assoc Lab Anim Sci.

[12] Morrison KE, Jašarević E, Howard CD, Bale TL (2020). It's the fiber, not the fat: significant effects of dietary challenge on the gut microbiome. Microbiome.

[13] Nisoli E, Tonello C, Cardile A, Cozzi V, Bracale R, Tedesco L (2005). Calorie restriction promotes mitochondrial biogenesis by inducing the expression of eNOS. Science.

[14] Duffy PH, Lewis SM, Mayhugh MA, McCracken A, Thorn BT, Reeves PG (2002). Effect of the AIN-93M purified diet and dietary restriction on survival in Sprague-Dawley rats: Implications for Chronic Studies. J Nutr.

[15] Ingram DK, Anson RM, de Cabo R, Mamczarz J, Zhu M, Mattison J (2004). Development of calorie restriction mimetics as a prolongevity strategy. Ann N Y Acad Sci.

[16] Lewis SM, Johnson ZJ, Mayhugh MA, Duffy PH (2003). Nutrient intake and growth characteristics of male Sprague-Dawley rats fed AIN-93M purified diet or NIH-31 natural-ingredient diet in a chronic two-year study. Aging Clin Exp Res.

[17] Le Couteur DG, Solon-Biet SM, Cogger VC, Ribeiro R, de Cabo R, Raubenheimer D (2020). Branched chain amino acids, aging and age-related health. Ageing Res Rev.

[18] López-Domínguez JA, Ramsey JJ, Tran D, Imai DM, Koehne A, Laing ST (2015). The influence of dietary fat source on life span in calorie restricted mice. J Gerontol A Biol Sci Med Sci.

[19] Acosta-Rodríguez V, Rijo-Ferreira F, Izumo M, Xu P, Wight-Carter M, Green CB (2022). Circadian alignment of early onset caloric restriction promotes longevity in male C57BL/6J mice. Science.

[20] Guide for Authors (2001). The Journal of Nutrition. 2001;131(1):2–5.

