# Supplementary material for: Chow diet in mouse aging studies: nothing regular about it
Source: GeroScience. 2023 Apr 20;45(3):2079–84. doi: 10.1007/s11357-023-00775-9 (PMC10400503; doi:10.1007/s11357-023-00775-9)
Supplement: Supplementary file 1 — Supplementary file1 (PDF 1794 KB) [file 11357_2023_775_MOESM1_ESM.pdf]

Supplemental Data:
Diet Formulation PDFs for Manuscript

Manuscript Title:

Chow diet in mouse aging studies: nothing regular about it

Authors:

Jennifer Lee, Ph.D.[1*]

Chloe Purello, B.S.[1]

Sarah L. Booth, Ph.D [1,]

Brian Bennett, Ph.D [2,3]

Christopher D. Wiley, Ph.D [1]

Ron Korstanje, Ph.D [4]

Affiliations:

[1] Jean Mayer USDA Human Nutrition Research Center on Aging at Tufts University, Boston, MA

[2] Agricultural Research Service, US Department of Agriculture, Western Human Nutrition Research Center, Davis, CA, USA

[3] Department of Nutrition, University of California Davis, Davis, CA, USA

[4] The Jackson Laboratory, Bar Harbor, ME

Corresponding author:

*To whom correspondence should be addressed. Address: JM USDA HNRCA, 711 Washington St, Boston, MA 02111. Phone: (617) 556-3098. Email: jen.lee@tufts.edu

JL Rat & Mouse/Auto 6F EXT — 5K0G

GUARANTEED ANALYSIS

Crude protein not less than	18.00%
Crude fat not less than	6.00%
Crude fiber not more than	5.00%
Ash not more than	8.00%
Moisture not more than	12.00%
Sodium not more than (California requirement only)	0.65%

INGREDIENTS

Whole Wheat, Ground Corn, Ground Oats, Wheat Middlings, Dehulled Soybean Meal, Fish Meal, Soybean Oil, Dehydrated Alfalfa Meal, Corn Gluten Meal, Dicalcium Phosphate, Brewers Dried Yeast, Calcium Carbonate, Menadione Dimethylpyrimidinol Bisulfite (source of Vitamin K), Salt, DL-Methionine, Choline Chloride, Magnesium Oxide, Thiamine Mononitrate, Pyridoxine Hydrochloride, Cholecalciferol (Form of Vitamin D3), Vitamin A Acetate, Calcium Pantothenate, Ferrous Sulfate, Biotin, Manganous Oxide, Calcium Iodate, DL-Alpha Tocopheryl Acetate (Form of Vitamin E), Folic Acid, Vitamin B-12 Supplement, Riboflavin Supplement, Nicotinic Acid, Cobalt Carbonate, Zinc Oxide, Ferrous Carbonate, Copper Sulfate, Zinc Sulfate.

FEEDING DIRECTIONS

Feed ad libitum. Plenty of fresh, clean water should be available at all times.

CHEMICAL COMPOSITION [1]

Nutrients [2]

**Protein, %**	**19.3**
Arginine, %	1.09
Cystine, %	0.36
Glycine, %	0.96
Histidine, %	0.45
Isoleucine, %	0.79
Leucine, %	1.54
Lysine, %	0.97
Methionine, %	0.62
Phenylalanine, %	0.87
Tyrosine, %	0.58
Threonine, %	0.69
Tryptophan, %	0.23
Valine, %	0.91
Serine, %	0.95
Aspartic Acid, %	1.88
Glutamic Acid, %	4.40
Alanine, %	1.16
Proline, %	1.42
Taurine, %	0.05
**Fat (ether extract), %**	**6.1**
**Fat (acid hydrolysis), %**	**7.4**
Cholesterol, ppm	243
Linoleic Acid, %	2.66
Linolenic Acid, %	0.38
Arachidonic Acid, %	0.02
Omega-3 Fatty Acids, %	0.63
Total Saturated Fatty Acids, %	1.14
Total Monounsaturated Fatty Acids, %	1.27
**Fiber (crude), %**	**4.2**
Neutral Detergent Fiber[3], %	15.5
Acid Detergent Fiber[4], %	5.2
**Nitrogen-Free Extract (by difference), %**	**53.8**
Starch, %	35.6
Glucose, %	0.12
Fructose, %	0.17
Sucrose, %	0.69
Lactose, %	0.00
**Total Digestible Nutrients, %**	**76.4**
**Gross Energy, kcal/gm**	**4.18**
**Physiological Fuel Value[5], kcal/g**	**3.48**
**Metabolizable Energy, kcal/g**	**3.13**

Minerals

**Ash, %**	**6.5**
Calcium, %	1.32
Phosphorus, %	0.96
Phosphorus (non-phytate), %	0.71
Potassium, %	0.69
Magnesium, %	0.23
Sulfur, %	0.30
Sodium, %	0.29
Chloride, %	0.49
Fluorine, ppm	35.4
Iron, ppm	359
Zinc, ppm	84
Manganese, ppm	156
Copper, ppm	11
Cobalt, ppm	0.82
Iodine, ppm	2.14
Chromium (added), ppm	0.01
Selenium, ppm	0.39

Vitamins

Carotene, ppm	1.5
Vitamin K, ppm	20.0
Thiamin, ppm	79.4
Riboflavin, ppm	9.1
Niacin, ppm	87
Pantothenic Acid, ppm	37
Choline Chloride, ppm	2,001
Folic Acid, ppm	1.9
Pyridoxine, ppm	10.0
Biotin, ppm	0.3
Vitamin B-12, mcg/kg	51
Vitamin A, IU/g	20
Vitamin D-3 (added), IU/g	4.4
Vitamin E, IU/kg	45
Ascorbic Acid, ppm	0

Calories provided by:

Protein, %	22.206
Fat (ether extract), %	15.902
Carbohydrates, %	61.892

* Product Code

1. Formulation based on calculated values from the latest ingredient analysis information. Since nutrient composition of natural ingredients varies and some nutrient loss will occur due to manufacturing processes, analysis will differ accordingly.
2. Nutrients expressed as percent of ration except where otherwise indicated. Moisture content is assumed to be 10.0% for the purpose of calculations.
3. NDF = approximately cellulose, hemi-cellulose and lignin.
4. ADF = approximately cellulose and lignin.
5. Physiological Fuel Value (kcal/gm) - Sum of decimal fractions of protein, fat and carbohydrate (use Nitrogen Free Extract) x 4,9,4 kcal/gm respectively.

5/11/2017

LabDiet
www.labdiet.com

LabDiet® JL Rat and Mouse/Auto 6F 5K52*

DESCRIPTION

LabDiet® Rat and Mouse/Auto 6F 5K52 is an autoclavable, complete life-cycle diet formulated using managed formulation, delivering Constant Nutrition®. This is paired with the selection of highest quality ingredients to assure minimal inherent biological variation in long-term studies. LabDiet 5K52 is similar in formulation to 5K0Q, the primary diet used at The Jackson Laboratories. This diet does not contain added silicon dioxide which helps to reduce diet clumping post-autoclaving.

Features and Benefits

- Managed Formulation delivers Constant Nutrition®
- Excellent diet to meet the nutrient requirements for maintenance, breeding and lactation.
- Is similar in formulation to 5K0Q, the primary diet at The Jackson Laboratory. Specific information on strains fed can be obtained from The Jackson Laboratory.
- Standard cylinder shaped feed pellet.
- Does not contain silicon dioxide, an additive used to reduce diet clumping post-autoclaving.
- Fortified with extra nutrients to compensate for losses during processing and autoclaving.

Product Forms Available	Catalog #
• 5K52: Cylinder shaped pellet, 3/8" x 3/4"	0006666

Other Versions Available	Catalog #
• 5LG4 JL Rat and Mouse Irr 6F (25 lb Cap Sack®)	0038125
• 5LL4 JL Rat and Mouse Irr 6F 5 lb vacuum sealed, 6 per box (30 lb box)	0051675

GUARANTEED ANALYSIS

Crude protein not less than	18.00%
Crude fat not less than	6.00%
Crude fiber not more than	5.00%
Ash not more than	8.00%
Moisture not more than	12.00%

AUTOCLAVING SUGGESTIONS

During the autoclaving process, the pellets can beplaced on trays, in small bags or in larger bags, as long as the pellets are stacked no more than 3 inches high. When steam autoclaved, the pellets swell and exert force on adjacent pellets. If confined by a bag or container, the pressure causes sticking as greater polymerization of fibrous materials occurs under such conditions. ***Assay before and after autoclaving:*** Conditions of sterilization must be determined for each autoclaving unit. It is best to assay the diet before and after sterilization to determine nutrient losses. Microbiological studies should be done also to insure the degree of sterilization desired.

INGREDIENTS

Ground Wheat, Ground Corn, Ground Oats, Wheat Middlings, Fish Meal, Dehulled Soybean Meal, Soybean Oil, Dehydrated Alfalfa Meal, Corn Gluten Meal, Dicalcium Phosphate, Brewers Dried Yeast, Calcium Carbonate, Menadione Dimethylpyrimidinol Bisulfite (source of Vitamin K), Salt, DL-Methionine, Choline Chloride, Magnesium Oxide, Thiamine Mononitrate, Pyridoxine Hydrochloride, Vitamin D3 Supplement, Vitamin A Acetate, Calcium Pantothenate, Ferrous Sulfate, Biotin, Manganous Oxide, Calcium Iodate, Vitamin E Supplement, Folic Acid, Vitamin B-12 Supplement, Riboflavin Supplement, Nicotinic Acid, Zinc Oxide, Ferrous Carbonate, Copper Sulfate, Zinc Sulfate, Cobalt Carbonate.

FEEDING DIRECTIONS

Feed ad libitum to rodents. Provide plenty of fresh clean water at all times.

For information regarding shelf life please visit www.labdiet.com.

CHEMICAL COMPOSITION[1]

Nutrients[2]

**Protein, %**	**19.3**
Arginine, %	1.08
Cystine, %	0.35
Glycine, %	0.95
Histidine, %	0.45
Isoleucine, %	0.77
Leucine, %	1.53
Lysine, %	0.97
Methionine, %	0.63
Phenylalanine, %	0.86
Tyrosine, %	0.57
Threonine, %	0.69
Tryptophan, %	0.22
Valine, %	0.90
Serine, %	0.92
Aspartic Acid, %	1.84
Glutamic Acid, %	4.20
Alanine, %	1.17
Proline, %	1.40
Taurine, %	0.05
**Fat (ether extract), %**	**6.5**
**Fat (acid hydrolysis), %**	**7.8**
Cholesterol, ppm	247
Linoleic Acid, %	2.87
Linolenic Acid, %	0.40
Arachidonic Acid, %	0.02
Omega-3 Fatty Acids, %	0.63
Total Saturated Fatty Acids, %	1.17
Total Monounsaturated Fatty Acids, %	1.42
**Fiber (Crude), %**	**4.1**
Neutral Detergent Fiber[3], %	15.3
Acid Detergent Fiber[4], %	5.1
**Nitrogen-Free Extract (by difference), %**	**53.6**
Starch, %	35.9
Sucrose, %	0.79
**Total Digestible Nutrients,%**	**76.5**
**Gross Energy, kcal/gm**	**4.21**
**Physiological Fuel Value[5], kcal/gm**	**3.50**
**Metabolizable Energy, kcal/gm**	**3.14**

Minerals

**Ash, %**	**6.5**
Calcium, %	1.32
Phosphorus, %	0.95
Phosphorus (non-phytate), %	0.71
Potassium, %	0.66
Magnesium, %	0.22
Sulfur, %	0.30
Sodium, %	0.28
Chloride, %	0.49
Fluorine, ppm	36
Iron, ppm	360
Zinc, ppm	82
Manganese, ppm	160
Copper, ppm	10
Cobalt, ppm	0.82
Iodine, ppm	2.2
Chromium (added), ppm	0.01
Selenium, ppm	0.39

Vitamins

Carotene, ppm	1.5
Vitamin K, ppm	20
Thiamin, ppm	80
Riboflavin, ppm	9.0
Niacin, ppm	86
Pantothenic Acid, ppm	37
Choline Chloride, ppm	2000
Folic Acid, ppm	1.9
Pyridoxine, ppm	10
Biotin, ppm	0.30
B_{12}, mcg/kg	51
Vitamin A, IU/gm	20
Vitamin D_3 (added), IU/gm	4.4
Vitamin E, IU/kg	45
Ascorbic Acid, mg/gm	0.0

Calories provided by:

Protein, %	22.088
Fat (ether extract), %	16.618
Carbohydrates, %	61.294

*Product Code

1. Formulation based on calculated values from the latest ingredient analysis information. Since nutrient composition of natural ingredients varies and some nutrient loss will occur due to manufacturing processes, analysis will differ accordingly.
2. Nutrients expressed as percent of ration except where otherwise indicated. Moisture content is assumed to be 10.0% for the purpose of calculations.
3. NDF = approximately cellulose, hemi-cellulose and lignin.
4. ADF = approximately cellulose and lignin.
5. Physiological Fuel Value (kcal/gm) = Sum of decimal fractions of protein, fat and carbo- hydrate (use Nitrogen Free Extract) x 4,9,4 kcal/gm respectively.

NOTE: When assayed, actual levels may vary from calculated values.

LabDiet.
www.labdiet.com

Formulab Diet 5008*
Formulab Diet, Irradiated 5008C33*

DESCRIPTION

Formulab Diet is formulated to supply complete life-cycle nutrition for use in breeding colonies of rats and hamsters and many mouse strains. This diet is formulated using the unique and innovative concept of Constant Nutrition®, paired with the selection of highest quality ingredients to assure minimal inherent biological variation in long-term studies. The high energy, high quality protein formulation of this diet maximizes reproduction of rats and hamsters and is an excellent life-cycle diet for most rodents.

Features and Benefits

- Similar nutrient concentration to 5001, with higher energy content
- Maximizes reproductive performance of rats and hamsters; supports gestation and lactation simultaneously
- High quality animal protein added to create a superior balance of amino acids for optimum performance
- Formulated to feed rats, hamsters and many mouse strains
- Single product inventory
- Available in Irradiated or Non-Irradiated form
- ZDF rats were developed using 5008

Product Forms Available

- Oval pellet, 10 mm x 16 mm x 25 mm length (3/8"x5/8"x1")
  - Non-Irradiated available in 15 kg or 50 lb paper sacks
  - Irradiated available in 25 lb paper sacks
- Meal (ground pellets), special order

GUARANTEED ANALYSIS

Crude protein not less than	23.0%
Crude fat not less than	6.5%
Crude fiber not more than	4.0%
Ash not more than	8.0%
Added minerals not more than	2.5%

INGREDIENTS

Ground corn, dehulled soybean meal, ground wheat, fish meal, wheat middlings, porcine animal fat preserved with BHA, cane molasses, brewers dried yeast, porcine meat meal, wheat germ, ground oats, dried beet pulp, dehydrated alfalfa meal, calcium carbonate, dried whey, salt, menadione dimethylpyrimidinol bisulfite, choline chloride, cholecalciferol, vitamin A acetate, pyridoxine hydrochloride, dl-alpha tocopheryl acetate, thiamin mononitrate, folic acid, DL-methionine, nicotinic acid, calcium pantothenate, riboflavin, vitamin B_{12} supplement, manganous oxide, zinc oxide, ferrous carbonate, copper sulfate, zinc sulfate, calcium iodate, cobalt carbonate.

FEEDING DIRECTIONS

Plenty of fresh, clean water should be available to the animals at all times.

Rats- All rats will eat varying amounts of feed depending on their genetic origin. Larger strains will eat between 15-30 grams per day. Smaller strains will eat between 12-15 grams per day. Feeders in rat cages should be designed to hold two to three days supply of feed at one time.

Mice-Adult mice will eat 4 to 5 grams of pelleted ration daily. Some of the larger strains may eat as much as 8 grams per day per animal. Feed should be available on a free choice basis in wire feeders above the floor of the cage.

Hamsters-Adults will eat 10 to 14 grams per day.

CHEMICAL COMPOSITION[1]

Nutrients[2]

**Protein, %**	**23.5**
Arginine, %	1.44
Cystine, %	0.35
Glycine, %	1.23
Histidine, %	0.58
Isoleucine, %	1.20
Leucine, %	1.87
Lysine, %	1.40
Methionine, %	0.43
Phenylalanine, %	1.08
Tyrosine, %	0.66
Threonine, %	0.90
Tryptophan, %	0.28
Valine, %	1.19
Serine, %	1.20
Aspartic Acid, %	2.60
Glutamic Acid, %	4.77
Alanine, %	1.39
Proline, %	1.63
Taurine, %	0.02
**Fat (ether extract), %**	**6.5**
**Fat (acid hydrolysis), %**	**7.5**
Cholesterol, ppm	280
Linoleic Acid, %	1.37
Linolenic Acid, %	0.09
Arachidonic Acid, %	0.01
Omega-3 Fatty Acids, %	0.29
Total Saturated Fatty Acids, %	2.51
Total Monounsaturated Fatty Acids, %	2.32
**Fiber (Crude), %**	**3.8**
Neutral Detergent Fiber[3], %	11.3
Acid Detergent Fiber[4], %	4.0
**Nitrogen-Free Extract (by difference), %**	**49.4**
Starch, %	34.9
Glucose, %	0.22
Fructose, %	0.24
Sucrose, %	2.57
Lactose, %	0.39
**Total Digestible Nutrients,%**	**81.2**
**Gross Energy, kcal/gm**	**4.15**
**Physiological Fuel Value[5], kcal/gm**	**3.50**
**Metabolizable Energy, kcal/gm**	**3.31**

Minerals

**Ash, %**	**6.8**
Calcium, %	1.00
Phosphorus, %	0.65
Phosphorus (non-phytate), %	0.42
Potassium, %	1.10
Magnesium, %	0.20
Sulfur, %	0.24
Sodium, %	0.28
Chlorine, %	0.48
Fluorine, ppm	19
Iron, ppm	230
Zinc, ppm	73
Manganese, ppm	71
Copper, ppm	13
Cobalt, ppm	0.4
Iodine, ppm	0.8
Chromium, ppm	1.4
Selenium, ppm	0.23

Vitamins

Carotene, ppm	4.0
Vitamin K (as menadione),ppm	3.2
Thiamin Hydrochloride, ppm	16
Riboflavin, ppm	5.0
Niacin, ppm	109
Pantothenic Acid, ppm	15
Choline Chloride, ppm	2000
Folic Acid, ppm	3.0
Pyridoxine, ppm	6.0
Biotin, ppm	0.20
B_{12}, mcg/kg	20
Vitamin A, IU/gm	15
Vitamin D_3 (added), IU/gm	3.3
Vitamin E, IU/kg	55
Ascorbic Acid, mg/gm	—

Calories provided by:

Protein, %	26.849
Fat (ether extract), %	16.710
Carbohydrates, %	56.441

*Product Code

1. Formulation based on calculated values from the latest ingredient analysis information. Since nutrient composition of natural ingredients varies and some nutrient loss will occur due to manufacturing processes, analysis will differ accordingly.
2. Nutrients expressed as percent of ration except where otherwise indicated. Moisture content is assumed to be 10.0% for the purpose of calculations.
3. NDF = approximately cellulose, hemi-cellulose and lignin.
4. ADF = approximately cellulose and lignin.
5. Physiological Fuel Value (kcal/gm) = Sum of decimal fractions of protein, fat and carbohydrate (use Nitrogen Free Extract) x 4,9,4 kcal/gm respectively.

12/11/09

7017

NIH-31 Open Formula Mouse/Rat Sterilizable Diet

Product Description- 7017 is a fixed and open formula, autoclavable diet manufactured with high quality ingredients and designed to support maintenance, growth, and reproduction of rodents. 7017 is supplemented with additional vitamins to ensure nutritional adequacy after autoclaving. **Also available certified (7017C) and irradiated (7917).**

Ingredients (in descending order of inclusion)- Ground wheat, ground corn, ground oats, wheat middlings, fish meal, dehulled soybean meal, dehydrated alfalfa meal, corn gluten meal, soybean oil, dicalcium phosphate, brewers dried yeast, calcium carbonate, iodized salt, choline chloride, magnesium oxide, kaolin, ferrous sulfate, menadione sodium bisulfite complex (source of vitamin K activity), manganous oxide, thiamin mononitrate, niacin, calcium pantothenate, vitamin E acetate, vitamin A acetate, copper sulfate, zinc oxide, riboflavin, pyridoxine hydrochloride, vitamin B_{12} supplement, vitamin D_3 supplement, calcium iodate, biotin, folic acid, cobalt carbonate.

Standard Product Form: *Pellet*

Macronutrients		
Crude Protein	%	18.0
Fat (ether extract) [a]	%	4.7
Carbohydrate (available) [b]	%	46.5
Crude Fiber	%	4.0
Neutral Detergent Fiber [c]	%	13.6
Ash	%	6.2
Energy Density [d]	kcal/g (kJ/g)	3.0 (12.6)
Calories from Protein	%	24
Calories from Fat	%	14
Calories from Carbohydrate	%	62
**Minerals**		
Calcium	%	1.1
Phosphorus	%	1.0
Non-Phytate Phosphorus	%	0.7
Sodium	%	0.3
Potassium	%	0.6
Chloride	%	0.5
Magnesium	%	0.2
Zinc	mg/kg	47
Manganese	mg/kg	155
Copper	mg/kg	13
Iodine	mg/kg	2
Iron	mg/kg	270
Selenium	mg/kg	0.30
**Amino Acids**		
Aspartic Acid	%	1.5
Glutamic Acid	%	3.2
Alanine	%	1.1
Glycine	%	1.0
Threonine	%	0.7
Proline	%	1.5
Serine	%	0.9
Leucine	%	1.4
Isoleucine	%	0.8
Valine	%	0.8
Phenylalanine	%	0.8
Tyrosine	%	0.7
Methionine	%	0.4
Cystine	%	0.3
Lysine	%	0.8
Histidine	%	0.4
Arginine	%	1.0
Tryptophan	%	0.2

Vitamins		
Vitamin A [e, f]	IU/g	24.2
Vitamin D_3 [e, g]	IU/g	4.2
Vitamin E	IU/kg	41
Vitamin K_3 (menadione)	mg/kg	22
Vitamin B_1 (thiamin)	mg/kg	76
Vitamin B_2 (riboflavin)	mg/kg	7
Niacin (nicotinic acid)	mg/kg	87
Vitamin B_6 (pyridoxine)	mg/kg	9
Pantothenic Acid	mg/kg	39
Vitamin B_{12} (cyanocobalamin)	mg/kg	0.06
Biotin	mg/kg	0.30
Folate	mg/kg	2
Choline	mg/kg	1890
**Fatty Acids**		
C16:0 Palmitic	%	0.7
C18:0 Stearic	%	0.1
C18:1ω9 Oleic	%	1.0
C18:2ω6 Linoleic	%	1.9
C18:3ω3 Linolenic	%	0.2
Total Saturated	%	0.9
Total Monounsaturated	%	1.2
Total Polyunsaturated	%	2.1
**Other**		
Cholesterol	mg/kg	50

[a] Ether extract is used to measure fat in pelleted diets, while an acid hydrolysis method is required to recover fat in extruded diets. Compared to ether extract, the fat value for acid hydrolysis will be approximately 1% point higher.

[b] Carbohydrate (available) is calculated by subtracting neutral detergent fiber from total carbohydrates.

[c] Neutral detergent fiber is an estimate of insoluble fiber, including cellulose, hemicellulose, and lignin. Crude fiber methodology underestimates total fiber.

[d] Energy density is a calculated estimate of *metabolizable energy* based on the Atwater factors assigning 4 kcal/g to protein, 9 kcal/g to fat, and 4 kcal/g to available carbohydrate.

[e] Indicates added amount but does not account for contribution from other ingredients.

[f] 1 IU vitamin A = 0.3 μg retinol

[g] 1 IU vitamin D = 25 ng cholecalciferol

For nutrients not listed, insufficient data is available to quantify.

Nutrient data represent the best information available, calculated from published values and direct analytical testing of raw materials and finished product. Nutrient values may vary due to the natural variations in the ingredients, analysis, and effects of processing.

Teklad Diets are designed and manufactured for research purposes only.

© 2015 Envigo

0915

7012

Teklad LM-485 Mouse/Rat Sterilizable Diet

Product Description- 7012 is a fixed formula, autoclavable diet manufactured with high quality ingredients and designed to support growth and reproduction of rodents. Typical isoflavone concentrations (daidzein + genistein aglycone equivalents) range from 300 to 600 mg/kg. Absence of animal protein and fish meal minimizes the presence of nitrosamines. 7012 is supplemented with additional vitamins to ensure nutritional adequacy after autoclaving. **Also available certified (7012C) and irradiated (7912).**

Ingredients (in descending order of inclusion)- Ground corn, dehulled soybean meal, ground oats, wheat middlings, dehydrated alfalfa meal, soybean oil, corn gluten meal, calcium carbonate, dicalcium phosphate, brewers dried yeast, iodized salt, choline chloride, kaolin, magnesium oxide, L-lysine, DL-methionine, ferrous sulfate, menadione sodium bisulfite complex (source of vitamin K activity), vitamin E acetate, thiamin mononitrate, calcium pantothenate, manganous oxide, niacin, copper sulfate, zinc oxide, vitamin A acetate, pyridoxine hydrochloride, riboflavin, vitamin D_3 supplement, vitamin B_{12} supplement, folic acid, biotin, calcium iodate, cobalt carbonate.

Standard Product Form: Pellet

Macronutrients		
Crude Protein	%	19.1
Fat (ether extract) [a]	%	5.8
Carbohydrate (available) [b]	%	44.3
Crude Fiber	%	4.6
Neutral Detergent Fiber [c]	%	13.7
Ash	%	6.1
Energy Density [d]	kcal/g (kJ/g)	3.1 (13.0)
Calories from Protein	%	25
Calories from Fat	%	17
Calories from Carbohydrate	%	58
**Minerals**		
Calcium	%	1.0
Phosphorus	%	0.7
Non-Phytate Phosphorus	%	0.4
Sodium	%	0.3
Potassium	%	0.8
Chloride	%	0.5
Magnesium	%	0.2
Zinc	mg/kg	63
Manganese	mg/kg	93
Copper	mg/kg	23
Iodine	mg/kg	3
Iron	mg/kg	240
Selenium	mg/kg	0.16
**Amino Acids**		
Aspartic Acid	%	1.8
Glutamic Acid	%	2.8
Alanine	%	1.0
Glycine	%	0.8
Threonine	%	0.8
Proline	%	1.4
Serine	%	1.3
Leucine	%	1.7
Isoleucine	%	0.8
Valine	%	0.9
Phenylalanine	%	0.9
Tyrosine	%	0.8
Methionine	%	0.4
Cystine	%	0.3
Lysine	%	1.0
Histidine	%	0.5
Arginine	%	1.2
Tryptophan	%	0.3

Vitamins		
Vitamin A [e, f]	IU/g	30.0
Vitamin D_3 [e, g]	IU/g	2.4
Vitamin E	IU/kg	150
Vitamin K_3 (menadione)	mg/kg	80
Vitamin B_1 (thiamin)	mg/kg	95
Vitamin B_2 (riboflavin)	mg/kg	14
Niacin (nicotinic acid)	mg/kg	100
Vitamin B_6 (pyridoxine)	mg/kg	17
Pantothenic Acid	mg/kg	87
Vitamin B_{12} (cyanocobalamin)	mg/kg	0.09
Biotin	mg/kg	0.77
Folate	mg/kg	7
Choline	mg/kg	2200
**Fatty Acids**		
C16:0 Palmitic	%	0.6
C18:0 Stearic	%	0.2
C18:1ω9 Oleic	%	1.3
C18:2ω6 Linoleic	%	2.6
C18:3ω3 Linolenic	%	0.3
Total Saturated	%	0.8
Total Monounsaturated	%	1.3
Total Polyunsaturated	%	2.9
**Other**		
Cholesterol	mg/kg	--

[a] Ether extract is used to measure fat in pelleted diets, while an acid hydrolysis method is required to recover fat in extruded diets. Compared to ether extract, the fat value for acid hydrolysis will be approximately 1% point higher.

[b] Carbohydrate (available) is calculated by subtracting neutral detergent fiber from total carbohydrates.

[c] Neutral detergent fiber is an estimate of insoluble fiber, including cellulose, hemicellulose, and lignin. Crude fiber methodology underestimates total fiber.

[d] Energy density is a calculated estimate of *metabolizable energy* based on the Atwater factors assigning 4 kcal/g to protein, 9 kcal/g to fat, and 4 kcal/g to available carbohydrate.

[e] Indicates added amount but does not account for contribution from other ingredients.

[f] 1 IU vitamin A = 0.3 μg retinol

[g] 1 IU vitamin D = 25 ng cholecalciferol

For nutrients not listed, insufficient data is available to quantify.

Nutrient data represent the best information available, calculated from published values and direct analytical testing of raw materials and finished product. Nutrient values may vary due to the natural variations in the ingredients, analysis, and effects of processing.

Teklad Diets are designed and manufactured for research purposes only.

© 2015 Envigo

2018

Teklad Global 18% Protein Rodent Diet

Product Description- 2018 is a fixed formula, non-autoclavable diet manufactured with high quality ingredients and designed to support gestation, lactation, and growth of rodents. 2018 does not contain alfalfa, thus lowering the occurrence of natural phytoestrogens. Typical isoflavone concentrations (daidzein + genistein aglycone equivalents) range from 150 to 250 mg/kg. Exclusion of alfalfa reduces chlorophyll, improving optical imaging clarity. Absence of animal protein and fish meal minimizes the presence of nitrosamines. **Also available certified (2018C) and irradiated (2918). For autoclavable diet, refer to 2018S (Sterilizable) or 2018SX (Extruded & Sterilizable).**

Ingredients (in descending order of inclusion)- Ground wheat, ground corn, wheat middlings, dehulled soybean meal, corn gluten meal, soybean oil, calcium carbonate, dicalcium phosphate, brewers dried yeast, iodized salt, L-lysine, DL-methionine, choline chloride, kaolin, magnesium oxide, vitamin E acetate, menadione sodium bisulfite complex (source of vitamin K activity), manganous oxide, ferrous sulfate, zinc oxide, niacin, calcium pantothenate, copper sulfate, pyridoxine hydrochloride, riboflavin, thiamin mononitrate, vitamin A acetate, calcium iodate, vitamin B_{12} supplement, folic acid, biotin, vitamin D_3 supplement, cobalt carbonate.

Standard Product Form: Pellet

**Macronutrients**		
Crude Protein	%	18.6
Fat (ether extract) [a]	%	6.2
Carbohydrate (available) [b]	%	44.2
Crude Fiber	%	3.5
Neutral Detergent Fiber [c]	%	14.7
Ash	%	5.3
Energy Density [d]	kcal/g (kJ/g)	3.1 (13.0)
Calories from Protein	%	24
Calories from Fat	%	18
Calories from Carbohydrate	%	58
**Minerals**		
Calcium	%	1.0
Phosphorus	%	0.7
Non-Phytate Phosphorus	%	0.4
Sodium	%	0.2
Potassium	%	0.6
Chloride	%	0.4
Magnesium	%	0.2
Zinc	mg/kg	70
Manganese	mg/kg	100
Copper	mg/kg	15
Iodine	mg/kg	6
Iron	mg/kg	200
Selenium	mg/kg	0.23
**Amino Acids**		
Aspartic Acid	%	1.4
Glutamic Acid	%	3.4
Alanine	%	1.1
Glycine	%	0.8
Threonine	%	0.7
Proline	%	1.6
Serine	%	1.1
Leucine	%	1.8
Isoleucine	%	0.8
Valine	%	0.9
Phenylalanine	%	1.0
Tyrosine	%	0.6
Methionine	%	0.4
Cystine	%	0.3
Lysine	%	0.9
Histidine	%	0.4
Arginine	%	1.0
Tryptophan	%	0.2

**Vitamins**		
Vitamin A [e, f]	IU/g	15.0
Vitamin D_3 [e, g]	IU/g	1.5
Vitamin E	IU/kg	110
Vitamin K_3 (menadione)	mg/kg	50
Vitamin B_1 (thiamin)	mg/kg	17
Vitamin B_2 (riboflavin)	mg/kg	15
Niacin (nicotinic acid)	mg/kg	70
Vitamin B_6 (pyridoxine)	mg/kg	18
Pantothenic Acid	mg/kg	33
Vitamin B_{12} (cyanocobalamin)	mg/kg	0.08
Biotin	mg/kg	0.40
Folate	mg/kg	4
Choline	mg/kg	1200
**Fatty Acids**		
C16:0 Palmitic	%	0.7
C18:0 Stearic	%	0.2
C18:1ω9 Oleic	%	1.2
C18:2ω6 Linoleic	%	3.1
C18:3ω3 Linolenic	%	0.3
Total Saturated	%	0.9
Total Monounsaturated	%	1.3
Total Polyunsaturated	%	3.4
**Other**		
Cholesterol	mg/kg	--

[a] Ether extract is used to measure fat in pelleted diets, while an acid hydrolysis method is required to recover fat in extruded diets. Compared to ether extract, the fat value for acid hydrolysis will be approximately 1% point higher.

[b] Carbohydrate (available) is calculated by subtracting neutral detergent fiber from total carbohydrates.

[c] Neutral detergent fiber is an estimate of insoluble fiber, including cellulose, hemicellulose, and lignin. Crude fiber methodology underestimates total fiber.

[d] Energy density is a calculated estimate of *metabolizable energy* based on the Atwater factors assigning 4 kcal/g to protein, 9 kcal/g to fat, and 4 kcal/g to available carbohydrate.

[e] Indicates added amount but does not account for contribution from other ingredients.

[f] 1 IU vitamin A = 0.3 μg retinol

[g] 1 IU vitamin D = 25 ng cholecalciferol

For nutrients not listed, insufficient data is available to quantify.

Nutrient data represent the best information available, calculated from published values and direct analytical testing of raw materials and finished product. Nutrient values may vary due to the natural variations in the ingredients, analysis, and effects of processing.

Teklad Diets are designed and manufactured for research purposes only.

© 2015 Envigo

2016

Teklad Global 16% Protein Rodent Diet

Product Description- 2016 is a fixed formula, non-autoclavable diet manufactured with high quality ingredients and designed to support growth and maintenance. 2016 does not contain alfalfa or soybean meal, thus minimizing the occurrence of natural phytoestrogens. Typical isoflavone concentrations (daidzein + genistein aglycone equivalents) range from non-detectable to 20 mg/kg. Exclusion of alfalfa reduces chlorophyll, improving optical imaging clarity. Absence of animal protein and fish meal minimizes the presence of nitrosamines. **Also available certified (2016C) and irradiated (2916). For autoclavable diet, refer to 2016S (Sterilizable).**

Ingredients (in descending order of inclusion)- Ground wheat, ground corn, wheat middlings, corn gluten meal, calcium carbonate, dicalcium phosphate, soybean oil, brewers dried yeast, iodized salt, L-lysine, DL-methionine, choline chloride, magnesium oxide, vitamin E acetate, menadione sodium bisulfite complex (source of vitamin K activity), manganous oxide, ferrous sulfate, zinc oxide, niacin, calcium pantothenate, copper sulfate, pyridoxine hydrochloride, riboflavin, thiamin mononitrate, vitamin A acetate, calcium iodate, vitamin B_{12} supplement, folic acid, biotin, vitamin D_3 supplement, cobalt carbonate.

Standard Product Form: *Pellet*

Macronutrients		
Crude Protein	%	16.4
Fat (ether extract) [a]	%	4.0
Carbohydrate (available) [b]	%	48.5
Crude Fiber	%	3.3
Neutral Detergent Fiber [c]	%	15.2
Ash	%	4.9
Energy Density [d]	kcal/g (kJ/g)	3.0 (12.6)
Calories from Protein	%	22
Calories from Fat	%	12
Calories from Carbohydrate	%	66
**Minerals**		
Calcium	%	1.0
Phosphorus	%	0.7
Non-Phytate Phosphorus	%	0.4
Sodium	%	0.2
Potassium	%	0.6
Chloride	%	0.4
Magnesium	%	0.2
Zinc	mg/kg	70
Manganese	mg/kg	100
Copper	mg/kg	15
Iodine	mg/kg	6
Iron	mg/kg	200
Selenium	mg/kg	0.23
**Amino Acids**		
Aspartic Acid	%	1.0
Glutamic Acid	%	3.3
Alanine	%	0.9
Glycine	%	0.7
Threonine	%	0.6
Proline	%	1.5
Serine	%	0.8
Leucine	%	1.9
Isoleucine	%	0.7
Valine	%	0.8
Phenylalanine	%	0.9
Tyrosine	%	0.5
Methionine	%	0.3
Cystine	%	0.3
Lysine	%	0.8
Histidine	%	0.4
Arginine	%	0.8
Tryptophan	%	0.2

Vitamins		
Vitamin A [e, f]	IU/g	15.0
Vitamin D_3 [e, g]	IU/g	1.5
Vitamin E	IU/kg	110
Vitamin K_3 (menadione)	mg/kg	50
Vitamin B_1 (thiamin)	mg/kg	17
Vitamin B_2 (riboflavin)	mg/kg	15
Niacin (nicotinic acid)	mg/kg	75
Vitamin B_6 (pyridoxine)	mg/kg	18
Pantothenic Acid	mg/kg	33
Vitamin B_{12} (cyanocobalamin)	mg/kg	0.08
Biotin	mg/kg	0.40
Folate	mg/kg	4
Choline	mg/kg	1030
**Fatty Acids**		
C16:0 Palmitic	%	0.5
C18:0 Stearic	%	0.1
C18:1ω9 Oleic	%	0.7
C18:2ω6 Linoleic	%	2.0
C18:3ω3 Linolenic	%	0.1
Total Saturated	%	0.6
Total Monounsaturated	%	0.7
Total Polyunsaturated	%	2.1
**Other**		
Cholesterol	mg/kg	--

[a] Ether extract is used to measure fat in pelleted diets, while an acid hydrolysis method is required to recover fat in extruded diets. Compared to ether extract, the fat value for acid hydrolysis will be approximately 1% point higher.

[b] Carbohydrate (available) is calculated by subtracting neutral detergent fiber from total carbohydrates.

[c] Neutral detergent fiber is an estimate of insoluble fiber, including cellulose, hemicellulose, and lignin. Crude fiber methodology underestimates total fiber.

[d] Energy density is a calculated estimate of *metabolizable energy* based on the Atwater factors assigning 4 kcal/g to protein, 9 kcal/g to fat, and 4 kcal/g to available carbohydrate.

[e] Indicates added amount but does not account for contribution from other ingredients.

[f] 1 IU vitamin A = 0.3 μg retinol

[g] 1 IU vitamin D = 25 ng cholecalciferol

For nutrients not listed, insufficient data is available to quantify.

Nutrient data represent the best information available, calculated from published values and direct analytical testing of raw materials and finished product. Nutrient values may vary due to the natural variations in the ingredients, analysis, and effects of processing.

Teklad Diets are designed and manufactured for research purposes only.

© 2015 Envigo

8604

ENVIGO

Teklad Rodent Diet

Product Description- 8604 is a fixed formula, non-autoclavable diet manufactured with high quality ingredients and designed to support growth and reproduction of rodents. Typical isoflavone concentrations (daidzein + genistein aglycone equivalents) range from 350 to 650 mg/kg. **Also available certified (8728C).**

Ingredients (in descending order of inclusion)- Dehulled soybean meal, wheat middlings, flaked corn, ground corn, fish meal, cane molasses, ground wheat, dried whey, soybean oil, brewers dried yeast, dicalcium phosphate, calcium carbonate, iodized salt, choline chloride, kaolin, magnesium oxide, ferrous sulfate, vitamin E acetate, menadione sodium bisulfite complex (source of vitamin K activity), manganous oxide, copper sulfate, zinc oxide, niacin, thiamin mononitrate, vitamin A acetate, vitamin D_3 supplement, calcium pantothenate, pyridoxine hydrochloride, riboflavin, vitamin B_{12} supplement, calcium iodate, folic acid, biotin, cobalt carbonate.

Standard Product Form: Pellet

Macronutrients		
Crude Protein	%	24.3
Fat (ether extract) [a]	%	4.7
Carbohydrate (available) [b]	%	40.2
Crude Fiber	%	4.0
Neutral Detergent Fiber [c]	%	12.4
Ash	%	7.4
Energy Density [d]	kcal/g (kJ/g)	3.0 (12.6)
Calories from Protein	%	32
Calories from Fat	%	14
Calories from Carbohydrate	%	54
**Minerals**		
Calcium	%	1.4
Phosphorus	%	1.1
Non-Phytate Phosphorus	%	0.7
Sodium	%	0.3
Potassium	%	1.0
Chloride	%	0.5
Magnesium	%	0.3
Zinc	mg/kg	80
Manganese	mg/kg	100
Copper	mg/kg	25
Iodine	mg/kg	2
Iron	mg/kg	300
Selenium	mg/kg	0.34
**Amino Acids**		
Aspartic Acid	%	2.3
Glutamic Acid	%	4.1
Alanine	%	1.4
Glycine	%	1.3
Threonine	%	0.9
Proline	%	1.6
Serine	%	1.6
Leucine	%	1.9
Isoleucine	%	1.0
Valine	%	1.1
Phenylalanine	%	1.1
Tyrosine	%	0.9
Methionine	%	0.4
Cystine	%	0.4
Lysine	%	1.4
Histidine	%	0.6
Arginine	%	1.5
Tryptophan	%	0.3

Vitamins		
Vitamin A [e, f]	IU/g	12.6
Vitamin D_3 [e, g]	IU/g	2.4
Vitamin E	IU/kg	120
Vitamin K_3 (menadione)	mg/kg	40
Vitamin B_1 (thiamin)	mg/kg	27
Vitamin B_2 (riboflavin)	mg/kg	8
Niacin (nicotinic acid)	mg/kg	63
Vitamin B_6 (pyridoxine)	mg/kg	13
Pantothenic Acid	mg/kg	21
Vitamin B_{12} (cyanocobalamin)	mg/kg	0.05
Biotin	mg/kg	0.38
Folate	mg/kg	3
Choline	mg/kg	2530
**Fatty Acids**		
C16:0 Palmitic	%	0.7
C18:0 Stearic	%	0.1
C18:1ω9 Oleic	%	0.9
C18:2ω6 Linoleic	%	1.9
C18:3ω3 Linolenic	%	0.2
Total Saturated	%	0.9
Total Monounsaturated	%	1.1
Total Polyunsaturated	%	2.1
**Other**		
Cholesterol	mg/kg	50

[a] Ether extract is used to measure fat in pelleted diets, while an acid hydrolysis method is required to recover fat in extruded diets. Compared to ether extract, the fat value for acid hydrolysis will be approximately 1% point higher.

[b] Carbohydrate (available) is calculated by subtracting neutral detergent fiber from total carbohydrates.

[c] Neutral detergent fiber is an estimate of insoluble fiber, including cellulose, hemicellulose, and lignin. Crude fiber methodology underestimates total fiber.

[d] Energy density is a calculated estimate of *metabolizable energy* based on the Atwater factors assigning 4 kcal/g to protein, 9 kcal/g to fat, and 4 kcal/g to available carbohydrate.

[e] Indicates added amount but does not account for contribution from other ingredients.

[f] 1 IU vitamin A = 0.3 μg retinol

[g] 1 IU vitamin D = 25 ng cholecalciferol

For nutrients not listed, insufficient data is available to quantify.

Nutrient data represent the best information available, calculated from published values and direct analytical testing of raw materials and finished product. Nutrient values may vary due to the natural variations in the ingredients, analysis, and effects of processing.

Teklad Diets are designed and manufactured for research purposes only.

SGS

© 2015 Envigo

0915

PicoLab® Rodent Diet 20 — 5053*

DESCRIPTION

PicoLab® Rodent Diet 20 is a 20% protein diet formulated for rat, hamster and mouse breeding colonies. This diet is formulated using the unique and innovative concept of Constant Nutrition®, paired with the selection of highest quality ingredients to assure minimal inherent biological variation in long-term studies. Irradiation gives reliable microbial control and eliminates the need for autoclaving. Irradiation treatment and special 3-ply packaging provide virtually bacteria-free dietary control.

Features and Benefits

- Constant Nutrition® formula helps minimize nutritional variables
- Formulated with 20% protein
- High quality animal protein added to create a superior balance of amino acids for optimum performance
- Recommended for rat breeding colonies and mice not requiring a higher energy diet
- Precision processing and selection of highest quality ingredients assures Constant Nutrition® quality
- Irradiation gives reliable microbial control and eliminates the need for autoclaving

Product Forms Available

- Oval pellet, 10 mm x 16 mm x 25 mm length (3/8"x5/8"x1")
- Meal (ground pellets), special order

Other Versions Available

- 5061 Pico-Vac® Lab Rodent Diet

GUARANTEED ANALYSIS

Crude protein not less than	20.0%
Crude fat not less than	4.5%
Crude fiber not more than	6.0%
Ash not more than	7.0%

INGREDIENTS

Ground corn, dehulled soybean meal, wheat middlings, ground wheat, fish meal, cane molasses, wheat germ, dried beet pulp, brewers dried yeast, dehydrated alfalfa meal, ground oats, soybean oil, dried whey, calcium carbonate, salt, DL-methionine, menadione dimethylpyrimidinol bisulfite (vitamin K), choline chloride, pyridoxine hydrochloride, cholecalciferol, vitamin A acetate, dl-alpha tocopheryl acetate, biotin, thiamin mononitrate, folic acid, vitamin B_{12} supplement, nicotinic acid, riboflavin, calcium pantothenate, manganous oxide, zinc oxide, ferrous carbonate, copper sulfate, zinc sulfate, calcium iodate, cobalt carbonate, sodium selenite.

FEEDING DIRECTIONS

Feed ad libitum to rodents. Plenty of fresh, clean water should be available to the animals at all times.

Rats- All rats will eat varying amounts of feed depending on their genetic origin. Larger strains will eat up to 30 grams per day. Smaller strains will eat up to 15 grams per day. Feeders in rat cages should be designed to hold two to three days supply of feed at one time.

Mice-Adult mice will eat up to 5 grams of pelleted ration daily. Some of the larger strains may eat as much as 8 grams per day per animal. Feed should be available on a free choice basis in wire feeders above the floor of the cage.

Hamsters-Adults will eat up to 14 grams per day.

CHEMICAL COMPOSITION[1]

Nutrients[2]

**Protein, %**	**20.0**
Arginine, %	1.22
Cystine, %	0.28
Glycine, %	0.96
Histidine, %	0.50
Isoleucine, %	0.97
Leucine, %	1.56
Lysine, %	1.16
Methionine, %	0.70
Phenylalanine, %	0.90
Tyrosine, %	0.59
Threonine, %	0.77
Tryptophan, %	0.26
Valine, %	1.00
Serine, %	1.03
Aspartic Acid, %	2.19
Glutamic Acid, %	4.34
Alanine, %	1.15
Proline, %	1.47
Taurine, %	0.02
**Fat (ether extract), %**	**5.0**
**Fat (acid hydrolysis), %**	**5.6**
Cholesterol, ppm	141
Linoleic Acid, %	2.19
Linolenic Acid, %	0.26
Arachidonic Acid, %	<0.01
Omega-3 Fatty Acids, %	0.33
Total Saturated Fatty Acids, %	0.93
Total Monounsaturated Fatty Acids, %	0.99
**Fiber (Crude), %**	**4.7**
Neutral Detergent Fiber[3], %	16.4
Acid Detergent Fiber[4], %	6.0
**Nitrogen-Free Extract (by difference), %**	**52.9**
Starch, %	33.9
Glucose, %	0.19
Fructose, %	0.23
Sucrose, %	3.18
Lactose, %	1.34
**Total Digestible Nutrients,%**	**76.2**
**Gross Energy, kcal/gm**	**4.07**
**Physiological Fuel Value[5], kcal/gm**	**3.41**
**Metabolizable Energy, kcal/gm**	**3.07**

Minerals

**Ash, %**	**6.1**
Calcium, %	0.81
Phosphorus, %	0.63
Phosphorus (non-phytate), %	0.33
Potassium, %	1.07
Magnesium, %	0.22
Sulfur, %	0.34
Sodium, %	0.30
Chlorine, %	0.51
Fluorine, ppm	10
Iron, ppm	220
Zinc, ppm	87
Manganese, ppm	85
Copper, ppm	13
Cobalt, ppm	0.71
Iodine, ppm	0.97
Chromium, ppm	0.81
Selenium, ppm	0.30

Vitamins

Carotene, ppm	1.5
Vitamin K (as menadione),ppm	3.3
Thiamin Hydrochloride, ppm	17
Riboflavin, ppm	8.0
Niacin, ppm	90
Pantothenic Acid, ppm	17
Choline Chloride, ppm	2000
Folic Acid, ppm	3.0
Pyridoxine, ppm	9.6
Biotin, ppm	0.30
B_{12}, mcg/kg	51
Vitamin A, IU/gm	15
Vitamin D_3 (added), IU/gm	2.2
Vitamin E, IU/kg	99
Ascorbic Acid, mg/gm	—

Calories provided by:

Protein, %	24.651
Fat (ether extract), %	13.205
Carbohydrates, %	62.144

*Product Code

1. Formulation based on calculated values from the latest ingredient analysis information. Since nutrient composition of natural ingredients varies and some nutrient loss will occur due to manufacturing processes, analysis will differ accordingly.
2. Nutrients expressed as percent of ration except where otherwise indicated. Moisture content is assumed to be 10.0% for the purpose of calculations.
3. NDF = approximately cellulose, hemi-cellulose and lignin.
4. ADF = approximately cellulose and lignin.
5. Physiological Fuel Value (kcal/gm) = Sum of decimal fractions of protein, fat and carbohydrate (use Nitrogen Free Extract) x 4,9,4 kcal/gm respectively.

LabDiet
www.labdiet.com

PicoLab® Mouse Diet 20 5058*

DESCRIPTION

PicoLab® Mouse Diet 20 is a Constant Nutrition® formulation providing 20% protein for mouse colonies that require extra levels of energy needed for maximum production in post-partum breeding. Irradiation treatment and special 4-ply packaging provide virtually bacteria-free dietary control.

Features and Benefits

- Formulated with 20% protein for mouse breeding colonies
- Precision processing and selection of highest quality ingredients assures Constant Nutrition® quality
- Designed to meet the energy needs of breeding mouse colonies, transgenic strains, and mice exposed to higher stress levels
- Irradiation gives reliable microbial control and eliminates the need for autoclaving

Product Forms Available

- Oval pellet, 10 mm x 16 mm x 25 mm length (3/8"x5/8"x1")
- Meal (ground pellets), special order

Other Versions Available

- 5062 Pico-Vac® Mouse Diet 20

GUARANTEED ANALYSIS

Crude protein not less than	20.0%
Crude fat not less than	9.0%
Crude fiber not more than	4.0%
Ash not more than	6.5%
Added minerals not more than	2.5%

INGREDIENTS

Ground wheat, ground corn, dehulled soybean meal, wheat germ, fish meal, brewers dried yeast, corn gluten meal, porcine animal fat preserved with BHA, soybean oil, calcium carbonate, salt, dicalcium phosphate, monocalcium phosphate, choline chloride, menadione dimethylpyrimidinol bisulfite, DL-methionine, vitamin A acetate, cholecalciferol, pyridoxine hydrochloride, dried whey, folic acid, dl-alpha tocopheryl acetate, biotin, thiamin mononitrate, calcium pantothenate, lecithin, riboflavin, nicotinic acid, casein, vitamin B_{12} supplement, manganous oxide, zinc oxide, ferrous carbonate, copper sulfate, zinc sulfate, calcium iodate, cobalt carbonate, sodium selenite.

FEEDING DIRECTIONS

Feed ad libitum to mice. Plenty of fresh, clean water should be available to the animals at all times.

Mice-Adult mice will eat up to 5 grams of pelleted ration daily. Some of the larger strains may eat as much as 8 grams per day per animal. Feed should be available on a free choice basis in wire feeders above the floor of the cage.

CHEMICAL COMPOSITION[1]

Nutrients[2]

**Protein, %**	**21.8**
Arginine, %	1.15
Cystine, %	0.31
Glycine, %	0.93
Histidine, %	0.50
Isoleucine, %	1.02
Leucine, %	1.82
Lysine, %	1.13
Methionine, %	0.67
Phenylalanine, %	0.97
Tyrosine, %	0.64
Threonine, %	0.79
Tryptophan, %	0.25
Valine, %	1.03
Serine, %	1.07
Aspartic Acid, %	2.13
Glutamic Acid, %	4.47
Alanine, %	1.34
Proline, %	1.54
Taurine, %	0.02
**Fat (ether extract), %**	**9.0**
**Fat (acid hydrolysis), %**	**9.1**
Cholesterol, ppm	200
Linoleic Acid, %	2.32
Linolenic Acid, %	0.21
Arachidonic Acid, %	0.02
Omega-3 Fatty Acids, %	0.32
Total Saturated Fatty Acids, %	2.72
Total Monounsaturated Fatty Acids, %	2.88
**Fiber (Crude), %**	**2.2**
Neutral Detergent Fiber[3], %	10.8
Acid Detergent Fiber[4], %	3.0
**Nitrogen-Free Extract (by difference), %**	**51.8**
Starch, %	39.3
Glucose, %	0.16
Fructose, %	0.16
Sucrose, %	0.71
Lactose, %	0.78
**Total Digestible Nutrients,%**	**85.3**
**Gross Energy, kcal/gm**	**4.60**
**Physiological Fuel Value[5], kcal/gm**	**3.75**
**Metabolizable Energy, kcal/gm**	**3.56**

Minerals

**Ash, %**	**5.0**
Calcium, %	0.81
Phosphorus, %	0.60
Phosphorus (non-phytate), %	0.33
Potassium, %	0.70
Magnesium, %	0.16
Sulfur, %	0.27
Sodium, %	0.25
Chlorine, %	0.42
Fluorine, ppm	12
Iron, ppm	200
Zinc, ppm	120
Manganese, ppm	120
Copper, ppm	17
Cobalt, ppm	0.55
Iodine, ppm	1.5
Chromium, ppm	0.56
Selenium, ppm	0.30

Vitamins

Carotene, ppm	Trace
Vitamin K (as menadione),ppm	3.1
Thiamin Hydrochloride, ppm	15
Riboflavin, ppm	8.0
Niacin, ppm	90
Pantothenic Acid, ppm	21
Choline Chloride, ppm	2200
Folic Acid, ppm	2.9
Pyridoxine, ppm	9.6
Biotin, ppm	0.30
B_{12}, mcg/kg	51
Vitamin A, IU/gm	15
Vitamin D_3 (added), IU/gm	3.3
Vitamin E, IU/kg	57
Ascorbic Acid, mg/gm	—

Calories provided by:

Protein, %	23.189
Fat (ether extract), %	21.635
Carbohydrates, %	55.176

*Product Code

1. Formulation based on calculated values from the latest ingredient analysis information. Since nutrient composition of natural ingredients varies and some nutrient loss will occur due to manufacturing processes, analysis will differ accordingly.
2. Nutrients expressed as percent of ration except where otherwise indicated. Moisture content is assumed to be 10.0% for the purpose of calculations.
3. NDF = approximately cellulose, hemi-cellulose and lignin.
4. ADF = approximately cellulose and lignin.
5. Physiological Fuel Value (kcal/gm) = Sum of decimal fractions of protein, fat and carbohydrate (use Nitrogen Free Extract) x 4,9,4 kcal/gm respectively.

PRODUCT DATA SHEET
Release date: August 2020

SAFE® A04

Ingredients

Barley, wheat, maize, soybean meal, wheat bran, hydrolyzed fish proteins, dicalcium phosphate, pre-mixture of minerals, calcium carbonate, pre-mixture of vitamins.

CENTESIMAL COMPOSITION

Cereals	84.1 %
Animal Proteins	4.0 %
Vegetal Proteins	8.0 %
Vitamins & Minerals	3.9 %

NUTRITIONAL COMPOSITION

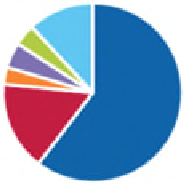

Nitrogen Free Extract	60.4 %
of which Starch	43.5 %
of which Sugars	3.2 %
Crude Protein	16.1 %
Crude Fat	3.1 %
Crude Ash	4.6 %
Crude Fiber	3.9 %
Moisture	11.9 %

ENERGY CONTENT

	MJ/kg	kcal/kg	%
ME Pig	13.2	3 145	
ME Atwater	14.0	3 339	
Energy from proteins	2.7	644	19.3
Energy from lipids	1.2	279	8.4
Energy from NFE	10.1	2 416	72.4

More information on energy calculation: www.safe-lab.com

Analysis End Product

TOTAL PER KG

AMINO ACIDS

Arginine	9 000 mg	Méthionine	2 800 mg
Cystine	2 500 mg	Tryptophane	1 900 mg
Lysine	7 200 mg	Glycine	8 100 mg

FATTY ACIDS

Palmitic acid	5 900 mg
Stearic acid	600 mg
Palmitoleic acid	150 mg
Oleic acid	4 800 mg
LA	15 000 mg
ALA	1 200 mg

MINERALS	END PRODUCT
Calcium	7 300 mg
Phosphorus	5 500 mg
Sodium	2 500 mg
Potassium	6 000 mg
Magnesium	1 600 mg
Manganese	70 mg
Iron	270 mg
Copper	16 mg
Zinc	55 mg
Chlorine	4 000 mg

VITAMINS	END PRODUCT
Vitamin A	7 500 IU
Vitamin D3	1 000 IU
Vitamin E	30 IU
Vitamin K3	2.5 mg
Vitamin B1	5.0 mg
Vitamin B2	6.5 mg
Vitamin B3	70 mg
Vitamin B5	10 mg
Vitamin B6	3.0 mg
Vitamin B9	0.35 mg
Vitamin B12	0.010 mg
Biotin	0.080 mg
Choline	1 600 mg

For the welfare of animals SAFE® bedding and environmental enrichment such as SAFE® block gnawing logs and SAFE® nesting materials should be available in the cage.

The values of the end products are given as indication only and have no contractual value. They are calculated averages of product analysis results before irradiation and autoclaving. Depending on production conditions, storage and analytical methods variations may occur. An analysis is performed on request.

Produced in France

WORLDWIDE HEADQUARTERS
73494 Rosenberg (Germany)
service@safe-lab.com

DIETS PRODUCTION SITE
89290 Augy (France)
info@safe-lab.com

Bio◆Serv

Delivering Solutions...

◆ Nutritional ◆ Enrichment ◆ Medicated ◆ Special Needs

Nutritional Profile

Product# F3155 - Rodent Diet, AIN-93M, 1/2" Pellets
Product# F3198 - Rodent Diet, AIN-93M, Powder

Proximate Profile

Protein	%	12.6
Fat	%	4.1
Fiber	%	4.8
Ash	%	2.2
Moisture	%	<10
Carbohydrate	%	67.5

Caloric Profile

Protein	kcal/gm	0.51
Fat	kcal/gm	0.37
Carbohydrate	kcal/gm	2.70
**Total**	**kcal/gm**	**3.58**

Amino Acids

Alanine	gm/kg	3.2
Arginine	gm/kg	4.5
Aspartic Acid	gm/kg	7.9
Cystine	gm/kg	2.2
Glutamic Acid	gm/kg	24.9
Glycine	gm/kg	3.0
Histidine	gm/kg	3.4
Isoleucine	gm/kg	6.7
Leucine	gm/kg	10.2
Lysine	gm/kg	9.1
Methionine	gm/kg	3.1
Phenylalanine	gm/kg	5.5
Proline	gm/kg	12.6
Serine	gm/kg	7.0
Threonine	gm/kg	5.4
Tryptophan	gm/kg	1.4
Tyrosine	gm/kg	7.0
Valine	gm/kg	8.0

Carbohydrates

Monosaccharides	gm/kg	0.7
Disaccharides	gm/kg	120
Polysaccharides	gm/kg	549

Fatty Acids

C18:2 Linoleic	gm/kg	20.4
C18:3 Linolenic	gm/kg	2.7
Total Saturated	gm/kg	6.3
Total Monounsaturated	gm/kg	9.1
Total Polyunsaturated	gm/kg	23.1

Minerals

Calcium	gm/kg	5.0
Chloride	gm/kg	1.6
Copper	mg/kg	6.0
Chromium	mg/kg	1.0
Fluoride	mg/kg	1.0
Iodine	mg/kg	0.21
Iron	mg/kg	36.6
Magnesium	gm/kg	0.51
Manganese	mg/kg	10.5
Phosphorus	gm/kg	2.8
Potassium	gm/kg	3.6
Selenium	mg/kg	0.17
Sodium	mg/kg	1032
Sulfur	mg/kg	301
Zinc	mg/kg	35.4

Vitamins

Ascorbic Acid	mg/kg	0.0
Biotin	mg/kg	0.20
Choline	mg/kg	1028
Folic Acid	mg/kg	2.0
Niacin	mg/kg	30.0
Pantothenic Acid	mg/kg	14.7
Pyridoxine	mg/kg	5.8
Riboflavin	mg/kg	6.0
Thiamin	mg/kg	5.3
Vitamin A	IU/kg	4100
Vitamin B_{12}	mcg/kg	25
Vitamin D_3	IU/kg	1000
Vitamin E	IU/kg	79.9
Vitamin K_1 (Phylloquinone)	mg/kg	0.82

Ingredients

Corn Starch, Casein, Maltodextrin, Sucrose, Cellulose, Soybean Oil, Mineral Mix, Vitamin Mix, Choline Bitartrate, L-Cystine, tBHQ

These are typical amounts of nutrients calculated from available information. Actual assay results may vary.
For more information contact Jaime Lecker, Ph.D.
Phone: 800-996-9908 ext. 112 (U.S. and Canada)
908-996-2155 (International)
Email: jlecker@bio-serv.com.

Revised Date: 1/11

Copyright© 2015 Bio-Serv
All Rights Reserved

Bio Serv

Delivering Solutions...

Nutritional • Enrichment • Medicated • Special Needs

Nutritional Profile

Product# F3156 - Rodent Diet, AIN-93G, 1/2" Pellets
Product# F3197 - Rodent Diet, AIN-93G, Powder

Proximate Profile

Protein	%	18.1
Fat	%	7.1
Fiber	%	4.8
Ash	%	2.2
Moisture	%	<10
Carbohydrate	%	59.3

Caloric Profile

Protein	kcal/gm	0.72
Fat	kcal/gm	0.64
Carbohydrate	kcal/gm	2.37
**Total**	**kcal/gm**	**3.74**

Amino Acids

Alanine	gm/kg	4.6
Arginine	gm/kg	6.4
Aspartic Acid	gm/kg	11.2
Cystine	gm/kg	3.5
Glutamic Acid	gm/kg	35.6
Glycine	gm/kg	4.3
Histidine	gm/kg	4.8
Isoleucine	gm/kg	9.6
Leucine	gm/kg	14.6
Lysine	gm/kg	13.0
Methionine	gm/kg	4.5
Phenylalanine	gm/kg	7.8
Proline	gm/kg	18.0
Serine	gm/kg	10.0
Threonine	gm/kg	7.7
Tryptophan	gm/kg	2.0
Tyrosine	gm/kg	10.0
Valine	gm/kg	11.4

Carbohydrates

Monosaccharides	gm/kg	0.6
Disaccharides	gm/kg	120
Polysaccharides	gm/kg	468

Fatty Acids

C18:2 Linoleic	gm/kg	35.7
C18:3 Linolenic	gm/kg	4.8
Total Saturated	gm/kg	11.0
Total Monounsaturated	gm/kg	15.9
Total Polyunsaturated	gm/kg	40.4

Minerals

Calcium	gm/kg	5.1
Chloride	gm/kg	1.6
Copper	mg/kg	6.0
Chromium	mg/kg	1.0
Fluoride	mg/kg	1.0
Iodine	mg/kg	0.21
Iron	mg/kg	37.2
Magnesium	gm/kg	0.51
Manganese	mg/kg	10.5
Phosphorus	gm/kg	2.8
Potassium	gm/kg	3.6
Selenium	mg/kg	0.17
Sodium	mg/kg	1030
Sulfur	mg/kg	301
Zinc	mg/kg	37.7

Vitamins

Ascorbic Acid	mg/kg	0.0
Biotin	mg/kg	0.20
Choline	mg/kg	1028
Folic Acid	mg/kg	2.0
Niacin	mg/kg	30.0
Pantothenic Acid	mg/kg	14.7
Pyridoxine	mg/kg	5.8
Riboflavin	mg/kg	6.0
Thiamin	mg/kg	6.0
Vitamin A	IU/kg	4140
Vitamin B_{12}	mcg/kg	25
Vitamin D_3	IU/kg	1000
Vitamin E	IU/kg	83.5
Vitamin K_1 (Phylloquinone)	mg/kg	0.88

Ingredients

Corn Starch, Casein, Maltodextrin, Sucrose, Soybean Oil, Cellulose, Mineral Mix, Vitamin Mix, L-Cystine, Choline Bitartrate, tBHQ

These are typical amounts of nutrients calculated from available information. Actual assay results may vary.
For more information contact Jaime Lecker, Ph.D.
Phone: 800-996-9908 ext. 112 (U.S. and Canada)
908-996-2155 (International)
Email: jlecker@bio-serv.com.

Revised Date: 1/11

Copyright© 2015 Bio-Serv
All Rights Reserved
